# Vitamin E succinate exerts anti-tumour effects on human cervical cancer cells via the CD47-SIRPɑ pathway both *in vivo* and *in vitro*

**DOI:** 10.7150/jca.52315

**Published:** 2021-05-05

**Authors:** Xiaoli Huang, Markus Neckenig, Jintang Sun, Di Jia, Yu Dou, Dan Ai, Zhaodi Nan, Xun Qu

**Affiliations:** 1 Department of Nutrition, Qilu Hospital of Shandong University, School of Medicine, Cheeloo College of Medicine, Shandong University, Jinan, Shandong, China.; 2 School of Pharmaceutical Sciences, Shandong University, Jinan, Shandong, China.; 3 Institute of Basic Medical Sciences, Qilu Hospital of Shandong University, School of Medicine, Cheeloo College of Medicine, Shandong University, Jinan, Shandong, China.; 4 Department of Biochemistry, Qiqihar Medical University, Qiqihar, Heilongjiang, China.; 5 Department of Tissue Engineering and Regeneration, School and Hospital of Stomatology, Shandong University & Shandong Key Laboratory of Oral Tissue Regeneration & Shandong Engineering Laboratory for Dental Materials and Oral Tissue Regeneration, Jinan, Shandong, China.

**Keywords:** CD47, vitamin E succinate, cervical cancer cells, *vivo*, * vitro*

## Abstract

Vitamin E succinate (RRR-a-tocopheryl succinate, VES) acts as a potent agent for cancer therapy and has no toxic and side effects on normal tissue cells. However, the mechanism by which VES mediates the effects are not yet fully understood. Here, we hypothesised that VES mediates antitumour activity on human cervical cancer cells via the CD47-SIRPɑ pathway *in vivo* and *in vitro*. Results indicated that the human cervical cancer HeLa cells treated with VES were more efficiently engulfed by THP-1-derived macrophages. In response to VES, the protein expression of CD47 on cell membranes and the mRNA level of CD47 in different human cervical cancer cells significantly decreased. And the level of calreticulin (CRT) mRNA in the VES-treated cells increased. By contrast, CRT protein expression was not altered. miRNA-155, miRNA-133 and miRNA-326 were up-regulated in the VES-treated HeLa cells. Knocking down miRNA-155 and miRNA-133 by RNA interference increased CD47 protein expression in the VES-treated cells. *In vivo* efficacy was determined in BALB/C nude mice with HeLa xenografts. Results showed that VES reduced tumour growth, increased overall survival and inhibited CD47 in the tumour transcriptionally and translationally. Furthermore, inflammatory factors (TNF-α, IL-12, IFN-γ, IL-2 and IL-10) in the spleen were altered because of VES treatment. Our results suggest that VES-induced antitumour activity is coupled to the CD47-SIRPɑ pathway in human cervical HeLa cancer cells.

## Introduction

Cervical cancer is a common malignant tumour from which a good many women are likely to suffer at some stage in their lives with the fourth highest probability by comparison with the incidence rate of other tumours occurrent in women and the seventh most common cancer in the general population 1. Similarly to other years, in 2018, to give but one example, the global incidence of cervical cancer was as worryingly high as 569,000 cases with a mortality rate of 311,000 cases 2. Therefore, novel more effective treatment methods are urgently needed to improve the survival rate of patients with cervical cancer. Great advances in the treatment of various human tumours have been achieved through the development of therapies that aim at modulating the immune system 3. Immunotherapy aims to eliminate cancer cells not only via the adaptive immunity mediated by T cells but also by utilising innate immune cells, such as macrophages 4. Macrophages serve both as phagocytes and as antigen-presenting cells. Macrophages can act as effectors killing tumour cells either by physical engulfment or by cytotoxin release 5. The transmembrane protein CD47 has been found to be more highly expressed in a large number of malignant cells. Research has shown that the CD47/the signal regulatory protein alpha (SIRPα) pathway plays an important role in inhibiting the phagocytosis of target cells 6.

CD47 performs a range of important physiological functions, such as adaptive immunity, bone remodelling, cell adhesion, motility, proliferation and survival 7, 8. As to its role in tumour development, CD47, which is an ubiquitously expressed surface receptor in all types of cancers, acts as an antiphagocytic “don't eat me” signal, binding to SIRPα on the macrophages and dendritic cells 9. High expression of CD47 is associated with poor survival in many cancers. Therefore, blockage of the CD47/SIRPα axis is a successful strategy to target and limit cancer cells 10.

A number of previous studies by other groups had already established that Vitamin E succinate (RRR-α-tocopheryl succinate, VES), a derivative of natural vitamin E, has the capacity to inhibit the growth of various cancer cell types *in vitro* and *in vivo* 11, 12. Our own previous studies had revealed that VES triggers apoptosis and inhibits cell proliferation via several pathways in human gastric cancer cells 13-15. We also found that VES upregulates the TRAIL expression in human CD4+T cells. Importantly, our previous work had shown that the combination of human CD4+T cells and VES can induce a higher anti-cancer effect against human gastric cancer cells 16, implying that VES possibly suppresses the growth of malignant cells via the immune system. Therefore, we decided to examine the expression of CD47 specifically on the surface of cervical cancer cells. In the present study, our specific focus has been to investigate whether the CD47/SIRPα axis is involved in the VES-induced anti-tumour effect. And we are pleased to report our important finding that, through participation in cell-molecular pathways, VES does indeed have the capacity to decrease CD47 expression of human cervical cancer cells *in vitro* and *in vivo*. Moreover, we have found that exposure to VES greatly increases THP-1 macrophage-mediated phagocytosis of these tumour cells, thereby further elucidating the anticancer immune mechanism of VES. To the best of our knowledge, this study is the first one of its kind in that we have specifically investigated the role of the CD47/SIRPα axis in the VES-induced anti-tumour effect.

## Materials and Methods

### Cell culture

Human cervical cancer HeLa and CaSki cells and the promonocytic cell line THP-1 were maintained in RPMI 1640 medium, while human cervical cancer SiHa cells were maintained in α-MEM medium, which were supplemented with 10% foetal bovine serum and 1% penicillin-streptomycin double antibody with 5% CO_2_ at 37 °C. The cells were sub-cultured at 80% fusion degree with a proportion of 1:3. Three times, a known quantity of exponentially growing cancer cells were treated with a liquid sample of VES (Sigma, USA) solution of defined concentration for the indicated time periods. The three VES samples had concentrations of 5, 7.5 and 10 mg/mL VES respectively - each time dissolved in 0.1% ethanol as solvent. HeLa and THP-1 cells were obtained from the cell bank of the Chinese Academy of Sciences in Shanghai and the American Type Culture Collection, respectively. CaSki and SiHa cells were respectively obtained from Procell Life Science & Technology Co. Ltd, and Cancer Center Laboratory of Shandong University.

### Phagocytosis assay

For each well, 2×10^5^ THP-1 cells were differentiated with 100 ng/mL PMA for 48 h. Cells treated with 10 μg/mL VES for 24 h were fluorescently stained with 5 μmol/L 5- or 6-(N-succinimidyloxycarbonyl)-fluorescein 3ʹ,6ʹ-diacetate (CFSE) for 15 min. After treatment, THP-1 macrophages were incubated in serum-free medium for 2 h before adding 2×10^5^ CFSE-labelled HeLa cancer cells. The macrophages were repeatedly washed and subsequently imaged using an inverted microscope. Also, HeLa cell/THP-1 macrophage co-cultures were analysed by flow cytometry after the treatment with PE-conjugated anti-CD11c antibody.

### Quantitative real-time PCR assays

Total RNA was extracted from cells and tissues with TRIzol reagent (Beyotime, China) according to the manufacturer's protocol. cDNA was synthesised with the PrimeScript RT reagent kit (Western Biotechnology). Real-time quantitative PCR (qRT-PCR) analyses were performed with the SYBR Green I and Real-time PCR reagents (Western Biotechnology). The 25 μL reaction mixture included 0.5 μL of 20 × SYBR Green I buffer, 2 μL of the template, 1 μL of upstream and downstream specific primers, and 20.5 μL of deionised water. RT-PCR included initial denaturing for 4 min at 94 °C, 35 cycles at 94 °C for 20 s, 60 °C for 30 s, and 72 °C for 30 s. Primer sequences were as follows: β-actin (forward: GAGACCTTCAACACCCCAGC; reverse: ATGTCACGCACGATTTCCC), CD47 (forward: TGAAGTGGAAGTTGAACAAATCG; reverse: GCTTATCCATTTTCAAAGAGGC), calreticulin (CRT) (forward: CTCTGTCGGCCAGTTTCGAG; reverse: TGTATTCTGAGTCTCCGTGCAT), TNF-α (forward: CCCTCCAGAAAAGACACCATG; reverse: CACCCCGAAGTTCAGTAGACAG), IL-12 (forward: CAGCACTTCAGAATCACAACCA; reverse: TCATTTTCACTCTGTAAGGGTCTG), IFN-γ (forward: TCAACAACCCACAGGTCCAG; reverse: CGACTCCTTTTCCGCTTCC), IL-2 (forward: AGATGAACTTGGACCTCTGCG; reverse: CATCTCCTCAGAAAGTCCACCAC) and IL-10 (forward: GGACAACATACTGCTAACCGACTC; reverse: CCTGGGGCATCACTTCTACC). The relative quantitative value for each gene was determined to be 2-∆∆CT.

### Antibodies and flow cytometry analysis

Anti-CD47 mAb B6H12 and anti-CRT mAb were obtained from Abcam (USA). After treating human cervical cancer cells with VES, they were labelled with a mixture of fluorochrome-conjugated monoclonal antiCD47 antibody and antiCRT antibody of saturating concentration according to the manufacturer's protocol. FACS Calibur (BD Biosciences, San Jose, CA, USA) analysis was used.

### Western blotting

Equal amounts of protein were separated on 10% SDS-PAGE and transferred onto a nitrocellulose membrane. Immunoblotting was performed using CD47 and β-actin antibodies (Abcam, USA). The secondary antibodies used were Sheep anti-rabbit IgG (Sigma, USA). After washing with TBST, membranes were incubated with the secondary sheep anti-rabbit IgG (Sigma, USA) and detected with the Western Blue Stabilised Substrate.

### Transient transfections

HeLa cells were plated in 6-well culture plates at a density of 2×10^5^ and transfected with siRNA using Lipofectamine 2000 (Invitrogen) according to the manufacturer's protocol. The key steps in summary, for each well, 5 μl lipofectamine 2000 was diluted in 245 μl serum-free Opti-MEM^®^-1 medium (Gibco). This first mixture was carefully added to a solution containing 5 μl siRNA in 250 μl dilution medium. This resultant mixture solution was incubated for 20 min at room temperature and then gently dripped into the HeLa cells in antibiotic-free medium; 30 h after transfections, the cells were treated with VES for 24 h. The inhibitors were designed as follows: miRNA-155 inhibitor (5ʹ-ACCCCUAUCACGAUUAGCAUUAA-3ʹ), miRNA-133 inhibitor (5ʹ-CAGCUGGUUGAAGGGGACCAAA-3ʹ), miRNA-326 inhibitor (5ʹ-CUGGAGGAAGGGCCCAGAGG-3ʹ) and miRNA inhibitor NC (5ʹ-CAGUACUUUUGUGUAGUACAA-3ʹ).

### Xenograft studies of nude mice

Experiments were approved by the Ethics Committee of Qilu Hospital of Shandong University and conducted according to institutional guidelines for animal care. HeLa cancer cells (1×10^7^ cells) were suspended in 200 μl phosphate-buffered saline and then injected subcutaneously under the subcutaneous part of the armpit of five-week-old female BALB/C nude mice (Chongqing Tengxin Biotechnology Co., Ltd.). When the size of the xenograft reached approximately 4 mm × 4 mm (length × width), the mice were randomly divided into three groups (control, 40 mg/kg VES and 80 mg/kg VES, n = 8). VES was injected intraperitoneally once every two days for 28 days. After the experiment, nine mice were kept to monitor their survival time.

### Statistical analysis

All experiments were performed at least three times. Data are presented as mean values±SD. Statistical differences were evaluated by ANOVA. Values with P < 0.05 were considered statistically significant.

## Results

### VES induces phagocytosis of HeLa cancer cells by THP-1 macrophages

**Figure 1:**
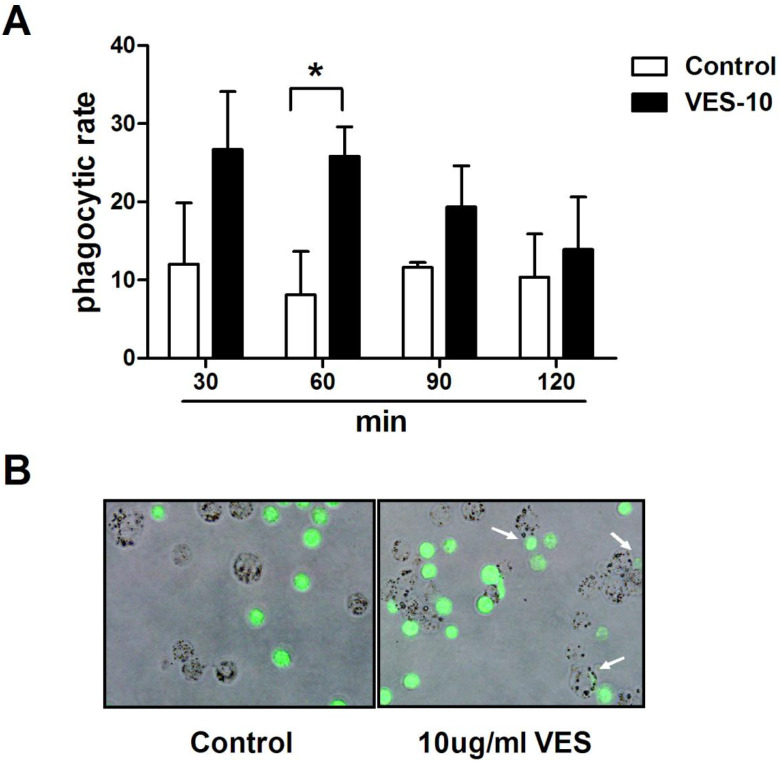
** VES induces phagocytosis of HeLa cancer cells by THP-1 macrophages. (A)** After treatment of 10 µg/mL VES for 24 h, the HeLa cancer cells were co-cultured with THP-1 macrophages for 30, 60, 90, 120 min. Phagocytosis of the tumour cells by macrophages was evaluated via flow cytometry after the treatment of PE-conjugated anti-CD11c antibody. Phagocytic rate was the number of phagocytosed cancer cells in 100 THP-1 macrophages.*P ≤ 0.05 compared with control. **(B)** Microscope image of THP-1 macrophages that phagocytosed HeLa cancer cells. HeLa cancer cells loaded with CFSE (green fluorescence) were co-cultured with THP-1 macrophages for 60 min. *Arrows*, individual phagocytosed HeLa cancer cells. The data are representative of at least 3 independent experiments.

After treatment with 10 μg/mL VES for 24 h, the HeLa cancer cells were co-cultured with THP-1 macrophages for 30, 60, 90, 120 min. The phagocytosis of the HeLa cells by macrophages was evaluated using fluorescence microscopy and flow cytometry. Figure 1 shows that exposure to VES greatly increases the degree of THP-1 macrophage-mediated phagocytosis of these tumour cells. This effect was found to be maximal when the tumour cells were co-cultured with THP-1 macrophages for 60 min. The phagocytic rate increased from 8.1% to 26.5%. In addition, the florescent images clearly confirmed that THP-1 macrophages had phagocytised the HeLa cancer cells.

### Effect of VES on CD47 and calreticulin expression of HeLa cancer cells

**Figure 2:**
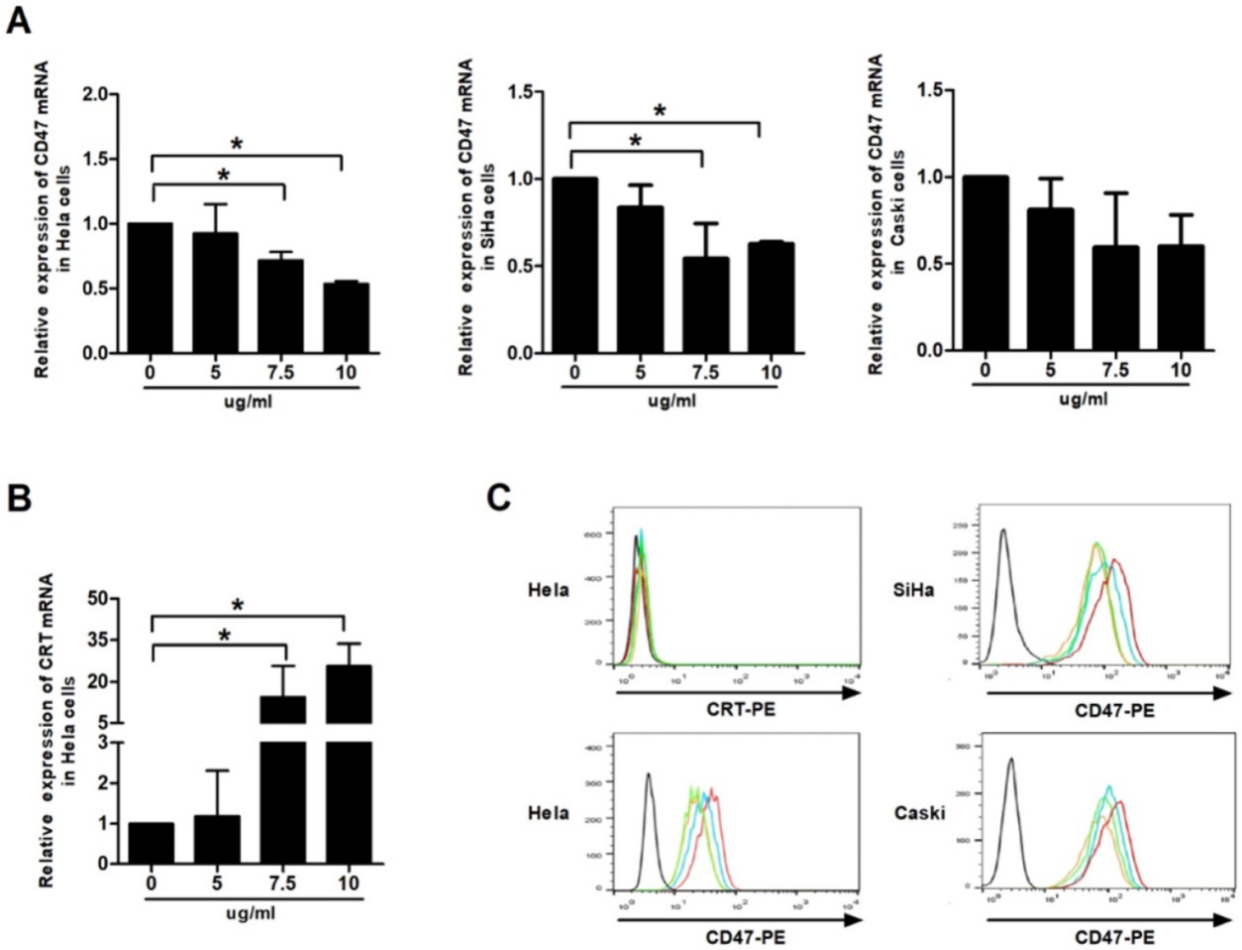
** Effect of VES on CD47 and calreticulin expression of** human cervical **cancer cells. (A)** HeLa, CaSki and SiHa cancer cells were treated with 5, 7.5 and 10 µg/mL VES for 12 h, and the expression of CD47 was examined by quantitative real-time PCR. **(B)** HeLa cancer cells were treated with 5, 7.5 and 10 µg/mL VES for 12 h, and the expression of calreticulin was examined by quantitative real-time PCR. **(C)** Cells were treated with 5, 7.5 and 10 µg/mL VES for 24 h and subjected to flow cytometry. Black: blank control, red: control group, blue: 5 µg/mL VES group, orange: 7.5 µg/mL VES group, green: 10 µg/mL VES group. The data are representative of at least 3 independent experiments. *P ≤ 0.05 compared with control.

CD47 and calreticulin levels were analysed using qRT-PCR and flow cytometry to examine whether phagocytosis of the human cervical cancer cells by THP-1 macrophages is mediated by phagocytic molecules. Our data showed that VES treatment produced down-regulation of the mRNA levels of CD47 in HeLa, CaSki and SiHa cells. Exposure of the HeLa cells to VES resulted in the up-regulation of the CRT mRNA. The concentration of surface CD47 protein decreased in the VES-treated HeLa, CaSki and SiHa cells. But CRT protein was not expressed on the surface of Hela cells both with and without the treatment of VES (Figs. 2A, 2B and 2C).

### miRNAs mediate CD47 expression in VES-treated HeLa cancer cells

**Figure 3:**
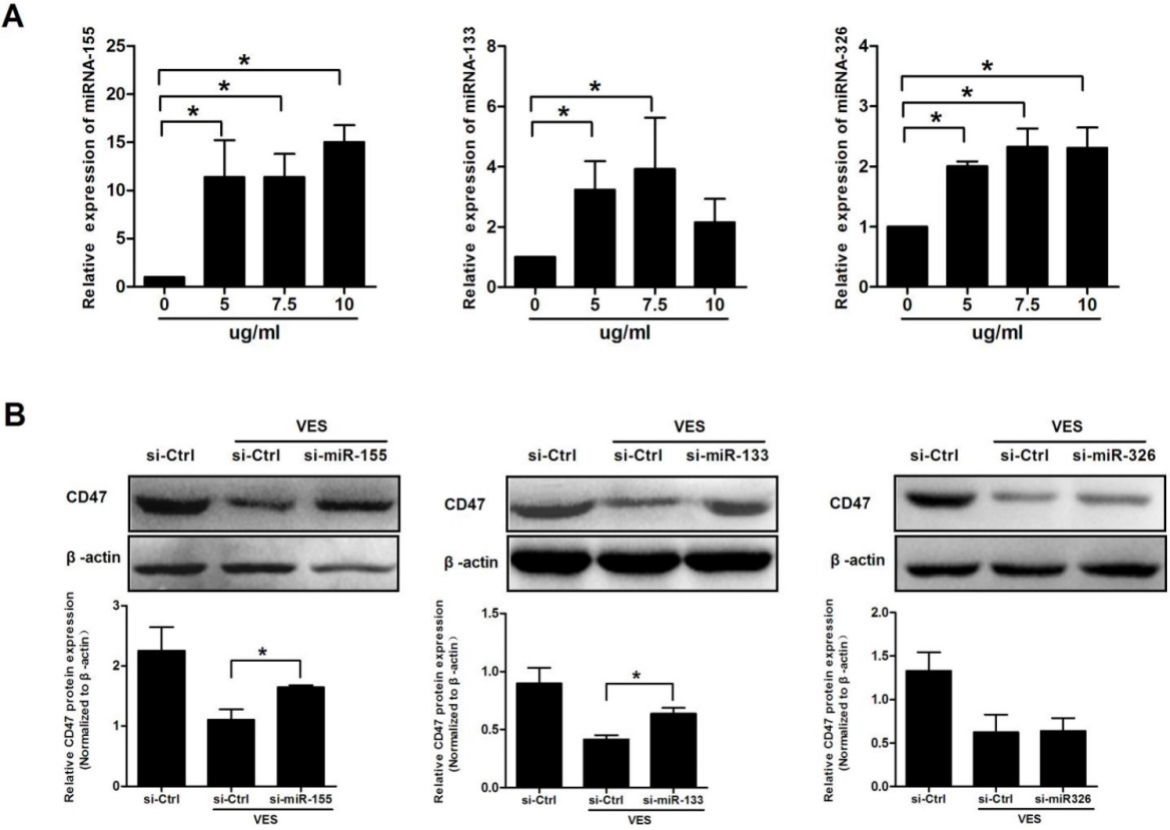
** miRNAs mediate CD47 expression in VES-treated HeLa cancer cells. (A)** HeLa cancer cells were treated with 5, 7.5 and 10 µg/mL VES for 3 h. miRNA-155 and miRNA-133 were examined via quantitative real-time PCR. After the cells were treated with VES for 6 h, miRNA-326 was measured. **(B)** After 30 h of transfection with the inhibitors of miRNA-133 and miRNA-326 and exposure of the cells to 7.5 µg/mL VES for 24 h, CD47 expression was examined via immunoblotting. After miRNA-155 siRNA-transfected cells were treated with 10 µg/mL VES for 24 h, CD47 level was measured. The data are representative of at least 3 independent experiments. *P ≤ 0.05 compared with control.

The HeLa cancer cells were treated with 5, 7.5 and 10 μg/mL VES for a time period of 3 h, and, separately, for 6 h. miRNA-155, miRNA-133 and miRNA-326 were analysed by qRT-PCR. Exposure of cells to VES induced miRNA-155, miRNA-133 and miRNA-326. Analysis revealed that miRNA-133 and miRNA-326 increased to the maximum in the cells that had been exposed to 7.5 μg/mL VES. By comparison, miRNA-155 increased to the maximum in the cells exposed to 10 μg/ml VES (Fig. 3A).

The VES-treated HeLa cells were cultured and transfected with the inhibitors of miRNA-155, miRNA-133 and miRNA-326 to assess the miRNAs on CD47 expression. CD47 levels were examined after 24 h of VES treatment using Western Blotting. Results showed that knockdown of miRNA-155 and miRNA-133 significantly reversed VES-induced decrease in CD47 protein expression. By contrast, the alteration of CD47 was not reversed by miRNA-326 down-regulation (Fig. 3B).

### VES inhibits the growth of HeLa xenografts in nude mice

**Figure 4:**
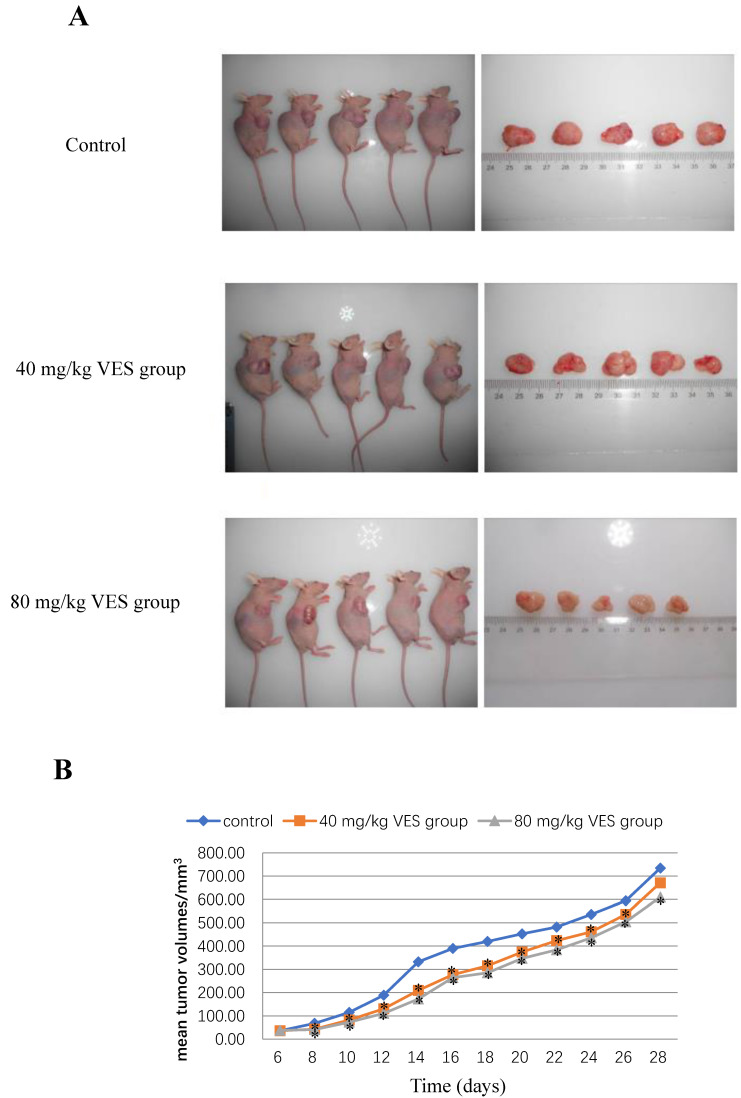
** VES inhibits the growth of HeLa xenografts in nude mice. (A)** Photographs of tumours from each treatment group excised from tumour-bearing mice. **(B)** Tumour volume changes after VES treatment on tumour-bearing mice (mean±SD, n = 5). *P ≤ 0.05 compared with control.

The reduction in tumour mass may be taken to be a reliable indicator of strong anti-tumour effects on the basis of accumulating and consistent evidence from a sufficiently large number of different and widely-accepted studies 17, 18. The anti-tumour effects of VES were evaluated in the HeLa tumour-bearing mice both (I) at a dose of 40 and (II) at 80 mg/kg. The mean tumour volumes in both VES treatments (I) and (II) were significantly lower than those in the control (P < 0.05 for both groups, Figs. 4A and 4B). Correspondingly, the mean tumour weights were lower for the VES groups (I) and (II) (0.78±0.04 and 0.72±0.03 g for 40 and 80 mg/kg VES, respectively) compared with those in the control group (0.98±0.12 g, P < 0.05, Table 1). The mean survival time of the control, the VES group (I) and the VES group (II) were 66±3.6, 79.7±2.08 and 86±2.65 days, respectively. Notably, the VES group(II) showed a substantially higher life extension value than the control (P < 0.05, Table 2).

### VES reduces CD47 expression in HeLa xenografts of nude mice

**Figure 5:**
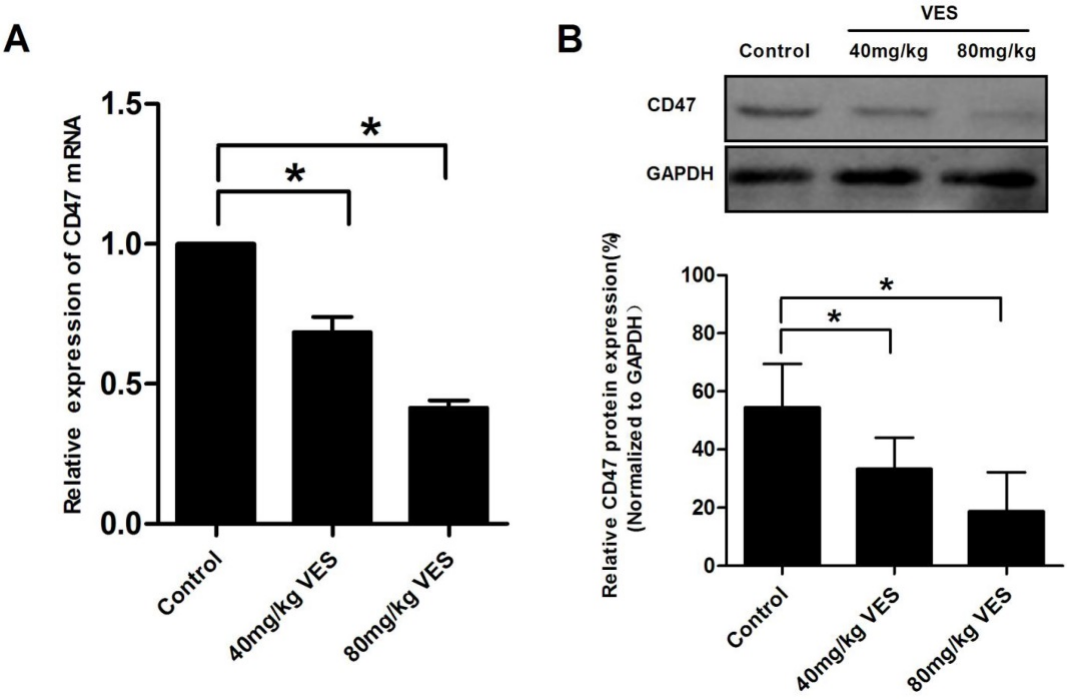
**VES reduces CD47 expression in HeLa xenografts of nude mice.** After the model of tumour-bearing mice was established, 40 and 80 mg/kg VES were injected intraperitoneally once every two days for 28 days. **(A)** CD47 mRNA in tumour excised from nude mice was detected by quantitative real-time PCR assays. β-Actin was used as a loading control. **(B)** CD47 protein in tumour excised from nude mice was detected by Western blotting. GAPDH was used as a loading control. Data are representative of at least three independent experiments. *P ≤ 0.05 compared with control. The data are representative of at least 3 independent experiments.

To assess the effect of VES on CD47 in HeLa xenografts of nude mice, we examined CD47 expression by means of qRT-PCR assays and Western Blotting. Our results showed that VES (I) and VES (II) concentrations significantly inhibited CD47 mRNA and protein expression in the tumours of the mice under examination (Fig. 5).

### VES alters the expression of inflammatory factors in the spleen of tumour-bearing mice

**Figure 6:**
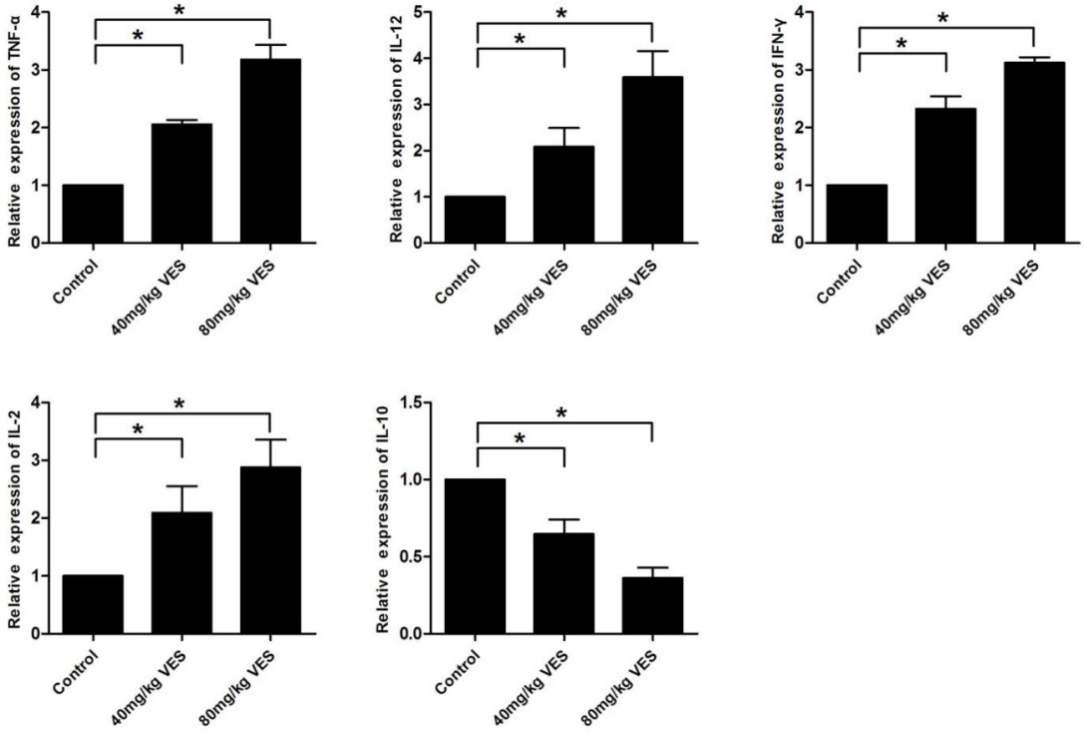
** VES alters the expression of inflammatory factors in the spleen of tumour-bearing mice.** After the model of tumour-bearing mice was established, 40 and 80 mg/kg VES were injected intraperitoneally once every two days for 28 days. Inflammatory factors TNF-α, IL-12, IFN-γ, IL-2 and IL-10 in the spleen of tumour-bearing mice were examined by quantitative real-time PCR assays. β-Actin was used as a loading control. Data are representative of at least three independent experiments. *P ≤ 0.05 compared with control. The data are representative of at least 3 independent experiments.

To further investigate the effects of VES on inflammatory factors in tumour-bearing mice, we examined TNF-α, IL-12, IFN-γ, IL-2 and IL-10 in the spleen by use of qRT-PCR assays. The pro-inflammatory cytokines (TNF-α, IL-12, IFN-γ and IL-2) increased in the spleen of tumour-bearing mice that had been treated with VES, whereas the anti-inflammatory cytokine IL-10 decreased transcriptionally (Fig. 6).

## Discussion

VES, a bio-active molecular compound, has been regarded for some time as a highly suitable chemotherapeutic agent, because of its anti-carcinogenic properties with no toxicity to normal cells or tissues 19, 20. VES has been shown to inhibit the tumour growth of xenografted human colon and breast tumour and allografted murine melanoma when administered i.p. 19, 21, 22. VES also suppresses chemical carcinogen-induced forestomach cancer in animal models 23. The present study has generated consistent data that convincingly show that intra-abdominal treatment with VES - in VES (I) and (II) concentrations (40 and 80 mg/kg) is a useful and effective method for significantly decreasing the weight and volume of tumour mass in HeLa tumour-bearing mice and for prolonging the survival time of the murine specimen.

Therefore: our results, which are consistent with those of previous studies, provide novel-additive, substantial and significant evidence in further support of the excellent anticancer activity of VES *in vivo*, and its broad anticancer effects to suppress cell growth via a variety of cell fates, including induction of apoptosis, DNA synthesis arrest and cellular differentiation 24-27. Our previous findings showed that endoplasmic reticulum stress, reactive oxygen species and intracellular TRAIL signalling are involved in VES-induced apoptosis in human gastric cancer cells 13, 14, 16, 28. Work by the group of Ramanathapuram provided evidence that VES treatment in combination with dendritic cells significantly increases the secretion of IFN-gamma compared with T cells from control and inhibits tumour growth in mice 29. VES has also been shown to modulate cell proliferation and cytokine production of murine EL-4 thymic lymphoma 30. Tomasetti and Neuzil confirmed that INF-gamma production by CD4+ and CD8+ T lymphocytes are induced by VES and TRAIL treatment in combination with dendritic cells 31. In agreement with these studies by other well-regarded research groups, our own previous results also demonstrated that VES up-regulates TRAIL expression in human CD4+ T cells, and that the combination of human CD4+ T cells and VES induces high anticancer activity of VES 16. Our preliminary data already indicated the possibility that VES may have antitumour effects by regulating the activity of the immune system. Now, with this present study, we are pleased to present new data clearly demonstrating that exposure to VES in 10 μg/mL concentration can greatly increase THP-1 macrophage-mediated phagocytosis of the HeLa cancer cells. Our present work substantially contributes to the view that the VES treatment constitutes a suitable method by substantially and therewith effectively increasing the phagocytosis rate of the HeLa cancer cells from 8.1% to 26.5% as shown by our experiments.

In recent years, escape from immunesurveillance is increasingly considered as a landmark event in cancer biology 32. The regulation of the anti-phagocytic signal CD47 is crucially important to surveillance against cancer cells 33. CD47 is a widely expressed transmembrane glycoprotein, which is also known as integrin-associated protein 34. CD47 is highly expressed in several human cancer types, such as non-Hodgkin's lymphomas, myeloid leukaemia, glioblastoma, leiomyosarcoma 35-38. High levels of CD47 expression have been found also in carcinomas, including breast, ovarian, bladder, colon, hepatocellular and cervical cancers 38, 39. CD47 performs its anti-phagocytic role by binding SIRPα on phagocytic cells, such as macrophages and dendritic cells, which makes SIRPα tyrosine phosphorylation, and by emitting inhibitory regulatory signals 8. The mechanism by which CD47 inhibits phagocytosis of tumour cells is well-understood thanks to robust and conclusive experimental and analytical work as reported in previous studies 6, 34. The CD47 blockade not only leads to macrophage surveillance of the innate immune system but also stimulates the adaptive immune system T-cell cytotoxicity 40. In addition, CD47 is involved in tumour progression, metastasis and outcome 41, 42. Therefore, CD47 should be regarded as a high-profile potential therapeutic target in cancer. In the present study, we have demonstrated that CD47 expression in the different human cervical cancer cells *in vitro* and in the HeLa xenografts of nude mice can be transcriptionally and translationally inhibited in response to VES. Therefore, it may be concluded that the CD47/SIRPα axis plays a very important functional role in the VES-induced anti-tumour activity on human cervical cancer cells.

Calreticulin, is an intracellular calcium-binding protein which is also known as an 'eat-me' signal. Calreticulin has been shown to be involved in anticancer immune response 43. CRT, which is predominantly localised in the lumen of the endoplasmic reticulum, can also translocate to cancer cell surfaces from where CRT provides the signal that is recognised by dendritic cells or other antigen-presenting cells 44. Binding of cell surface calreticulin to its macrophage receptor, a low-density lipoprotein-related protein, results in the phagocytosis of the target cell 43, 44. Our study has discovered that exposure of the HeLa cancer cells to VES induces CRT mRNA in the HeLa cells, whilst CRT protein is not expressed on the surface of Hela cells both with and without the treatment of VES. Hence, it can be reasoned that the CRT signal may not participate in the anticancer response of VES.

Previous research has shown that secretion of a large number of inflammatory cytokines is observed in tumour immune response and macrophage activation 45. Lian and co-workers reported that efficient silencing of CD47 and programmed death-ligand 1 increase the release of various cytokines, including IL-6 and IFN-γ, *in vitro* and *in vivo* 46. Vermeer and associates published a study on the effects of radiation-induced loss of cell surface CD47, namely an increase in phagocytosis of these cells by dendritic cells and also induction of IFN-γ and granzyme production from lymphocytes 47. Navarathna's group examined CD47^-/-^ mice and found that the serum inflammatory cytokines TNF-α, IL-6 and IL-10 were significantly elevated but IL-17 was decreased 48. In addition, Demeure and co-workers discovered that CD47 engagement suppresses cytokine production by dendritic cells, including IL-12, TNF-α, GM-CSF and IL-6 49. All of these studies provide strong, consistent and converging evidence that numerous cytokines are involved in the immune response mediated by CD47. Our results show that pro-inflammatory cytokines (TNF-α, IL-12, IFN-γ and IL-2) increase in the spleen of tumour-bearing mice when treated with VES, whereas the anti-inflammatory cytokine IL-10 decreased transcriptionally. Collectively, these data provide strong evidence that VES-induced antitumour activity on human cervical cancer cells is coupled to the CD47-SIRPα pathway.

However, the precise mechanisms by which CD47 expression is regulated remain unclear. Previous studies reported that miRNAs are involved in the regulation of CD47 expression. For example, Junker's group showed that three microRNAs, namely, miRNA-34a, miRNA-155 and miRNA-326, when up-regulated in active multiple sclerosis lesions, target the 3ʹ-untranslated region of CD47 and reduce CD47 in brain resident cells 50. Suzuki and co-workers have found that CD47 expression in oesophageal squamous cell carcinoma is directly suppressed by the miRNA-133a tumour suppressor 51. Vasques-Nóvoa and associates discovered that miRNA-155 up-regulation in septic myocardium directly targets CD47 in isolated cardiac microvascular endothelial cells 52. In agreement with this set of previous studies, the results of our own present study show that exposure of the HeLa cancer cells to VES induces miRNA-155, miRNA-133 and miRNA-326. We have found that, unlike miRNA-326 down-regulation, knockdown of miRNA-155 and miRNA-133 significantly reversed VES-induced decrease in CD47 protein expression. On the basis of these important findings, we therefore wish to propose that miRNA-155 and miRNA-133 are likely to contribute to VES-induced CD 47 down-regulation in human HeLa cancer cells.

In summary, we are delighted that our present study provides strong and consistent two-fold evidence for the first timeꓽfirstly that VES-induced anti-tumour activity on human cervical cancer cells is mediated, at least in part, by the CD47-SIRPα pathway *in vivo* and *in vitro*, and secondly that the phagocytosis of tumour cells by macrophages is implicated in this process. We are confident that this study constitutes a valuable and important contribution to advancing the present state of knowledge and that our work will help to further deepen the systematic understanding of the ways in which the CD47-SIRPα pathway plays a critical role in the regulation of anti-tumour activity in VES-exposed cells. We wish to share with the wider research community our view that thorough investigation of the mechanisms underlying VES-induced anticancer efficacy would be a praise-worthy goal of future research. Our own team certainly hopes and plans to contribute to these endeavours in the future.

## Figures and Tables

**Table 1:** Average weights of HeLa xenografts in nude mice from different groups (mean ± SE, n=5)

Group	Tumor weight (g)	Rate of tumor inhibition (%)
Control	0.98±0.12	-
40 mg/kg VES	0.78±0.04*	20.04
80 mg/kg VES	0.72±0.03*	26.07

Note: *P < 0.05 compared with the control group.

**Table 2:** The mean survival time of HeLa xenografts in nude mice from different groups (mean ± SE, n=3)

Group	Survival time (days)
Control	66±3.6
40 mg/kg VES	79.7±2.08*
80 mg/kg VES	86±2.65*

Note: *P < 0.05 compared with the control group.
